# MTAP-deficiency could predict better treatment response in advanced lung adenocarcinoma patients initially treated with pemetrexed-platinum chemotherapy and bevacizumab

**DOI:** 10.1038/s41598-020-57812-2

**Published:** 2020-01-21

**Authors:** Wang Jing, Hui Zhu, Wenjuan Liu, Xiaoyang Zhai, Hairong Tian, Jinming Yu

**Affiliations:** 1 The First Affiliated Hospital of Zhengzhou University, Department of Radiation Oncology, Zhengzhou, 450001 Henan Province China; 2 Shandong Cancer Hospital and Institute, Shandong First Medical University and Shandong Academy of Medical Science, Department of Radiation Oncology, Jinan, 250117 Shandong Province China; 3 Shandong Cancer Hospital and Institute, Shandong First Medical University and Shandong Academy of Medical Science, Shandong Provincial Key Laboratory of Radiation Oncology, Cancer Research Center, Jinan, 250117 Shandong Province China

**Keywords:** Non-small-cell lung cancer, Risk factors

## Abstract

To investigate the predictive value of methylthioadenosine phosphorylase (MTAP) on treatment response and survival in advanced lung adenocarcinoma. MTAP expression was detected by immunohistochemistry. Treatment response and survival were compared according to MTAP expression level. The results indicated MTAP-low expression was observed in 61.2% (101/165) of all patients. The objective response rate and disease control rate improved in the MTAP-low group (64.4% vs 46.9%, *p* = 0.035; 92.1% vs. 79.7%, *p* = 0.03; respectively). The median progression-free survival and survival time in the MTAP-low group were significantly lower than that in the MTAP-high group (8.1 vs. 13.1 months, *p* = 0.002; 22 vs. 32 months, *p* = 0.044). Multivariate analysis demonstrated that brain metastasis (HR 1.55, *p* = 0.046), thoracic radiation (HR 0.52, *p* = 0.026), and MTAP-low expression (HR 1.36, *p* = 0.038) were independent factors on survival. It is concluded that MTAP-low expression could predict improved treatment response but worsened survival in advanced lung adenocarcinoma.

## Introduction

Lung cancer is the leading cause of cancer-related death in China and worldwide, with lung adenocarcinoma as the predominant subtype of lung cancer, accounting for 66.2% in Asian populations^1–3^. Due to the massive population base and the lack of early screening in China, a majority of people were diagnosed initially with locally advanced or metastatic non-small cell lung cancer. Although approximately 50% of lung adenocarcinoma were EGFR-derived in Asia, a significant number of patients have no or unknown gene-mutation. Consequently, the combination of pemetrexed with platinum plus bevacizumab was still the first-line strategy for non-mutant lung adenocarcinoma based on the great efficacy of chemotherapy plus antiangiogenetic therapy of phase III BEYOND trial^4^. However, 36–46% of patients have no response or even progress to front-line treatment^5^. As a result, screening an efficient biomarker to predict chemo response is a priority.

An optimal marker should distinguish between tumor cells and normal cells and play a vital role in cellular metabolic pathways. Methylthioadenosine phosphorylase (MTAP), a purine metabolic enzyme, is abundant in normal tissues but deficient in 44% of lung adenocarcinoma^6^. MTAP cleaves methylthioadenosine (MTA) to adenine and 5-methythioribose-1-phosphate (MTR-1-P)^7^. Adenine is recruited as a substrate to synthesize adenosine monophosphate (AMP) via the *de novo* biosynthesis pathway, whereas MTR-1-P is used for methionine synthesis. Methionine is further recycled to synthesize MTA with the participation of folate, multiple synthases, and adenine metabolites, that forms a loop of purine metabolism^8,9^. Inhibition of MTAP decreased the level of adenine in A549 MTAP^−/−^ xenograft tumor significantly compared that in H358 MTAP^+/+^ model; moreover, the tumor growth in A549 MTAP^−/−^ xenograft mice was also inhibited dramatically by MTAP inhibitor in contrast to untreated mice^10^. Thus, MTAP plays a vital role in the salvage of purine and methionine to synthesize DNA. MTAP-deficient tumor cells are more sensitive to inhibitors of *de novo* purine synthesis than cells with intact MTAP^11^.

In clinical, blockades of *de novo* purine synthesis achieved favorable results in parts of MTAP-deficient tumors, but the role of MTAP expression still have some unclear in lung cancer. Pemetrexed is a well-known multitargeted antifolate that inhibits the *de novo* pathway of purine and pyrimidine synthesis^8^. A phase II trial indicated the response rate of pemetrexed for heavily pretreated MTAP-deficient advanced urothelial carcinoma was 66%^12^. However, regardless of the excellent blockades of purine synthesis on pemetrexed to MTAP-deficient lung adenocarcinoma cell lines in basic research, it is still unclear whether MTAP deficiency could predict the sensitivity of pemetrexed-based treatments in lung adenocarcinoma clinically. Therefore, we conducted this retrospective study to explore the correlation between MTAP expression and clinical outcomes in patients with advanced lung adenocarcinoma who received pemetrexed-based first-line chemotherapy.

## Results

### Patients

A total of 165 patients were reviewed in the present study. The clinical characteristics of the patients were shown in Table 1. The majority of patients were male (63.6%), former or current smoker (68.5%), and stage N2–3 (78.8%). The median age of the whole group was 59 years old (range, 32–76 years). The Karnofsky Performance Status score for all patients before initial treatment was not less than 70. Among them, 118 (71.5%) patients underwent gene mutant detection, while 39.4% of patients were with EGFR mutation. The median cycles of first-line chemotherapy plus bevacizumab were 4 (range, 1–6). Maintenance therapy with pemetrexed alone or pemetrexed plus bevacizumab was performed in 64 and 48 patients, respectively. The median cycles of maintenance therapy were 2 (range, 0–30). No patients underwent maintenance therapy with bevacizumab alone. In addition, a total of 96 (58.2%) patients were given consolidative thoracic radiation (TRT) with a median dose of 60 Gy (range, 50–60 Gy).

**Table 1:** Clinical pathological features of 165 patients with advanced-stage non-small cell lung cancer.

Characteristics	N (%)	MTAP expression, n (%)		*p*
		Low (n = 101)	High (n = 64)	
Gender				
Male	105 (63.6)	67 (66.3)	38 (59.4)	0.41
Female	60 (36.4)	34 (33.7)	26 (40.6)	
Age				
≥60	78 (47.3)	53 (52.5)	25 (39.1)	0.11
<60	87 (52.7)	48 (47.5)	39 (60.9)	
Smoking status				
Nonsmoker	52 (31.5)	28 (27.7)	24 (37.5)	0.23
Former/current smoker	113 (68.5)	73 (72.3)	40 (62.5)	
T stage				
T1-2	79 (47.9)	45 (44.6)	34 (53.1)	0.34
T3-4	86 (52.1)	56 (55.4)	30 (46.9)	
N stage				
N0-1	35 (21.2)	20 (19.8)	15 (23.4)	0.70
N2-3	130 (78.8)	81 (80.2)	49 (76.6)	
First-line therapy cycles				
4	115 (69.7)	73 (72.3)	42 (65.6)	0.36
6	50 (30.3)	28 (27.7)	22 (34.4)	
Thoracic radiation				
Yes	96 (58.2)	56 (55.4)	40 (62.5)	0.42
No	69 (41.8)	45 (44.6)	24 (37.5)	
EGFR mutations				
Wild-type	53 (32.1)	34 (33.7)	19 (29.7)	0.41
Mutation	65 (39.4)	42 (41.6)	23 (35.9)	
Unknown	47 (28.5)	25 (24.7)	22 (34.4)	
Recurrence				
Yes	79 (47.9)	55 (54.5)	24 (37.5)	0.04
No	86 (52.1)	46 (45.5)	40 (62.5)	

**Figure 1:**
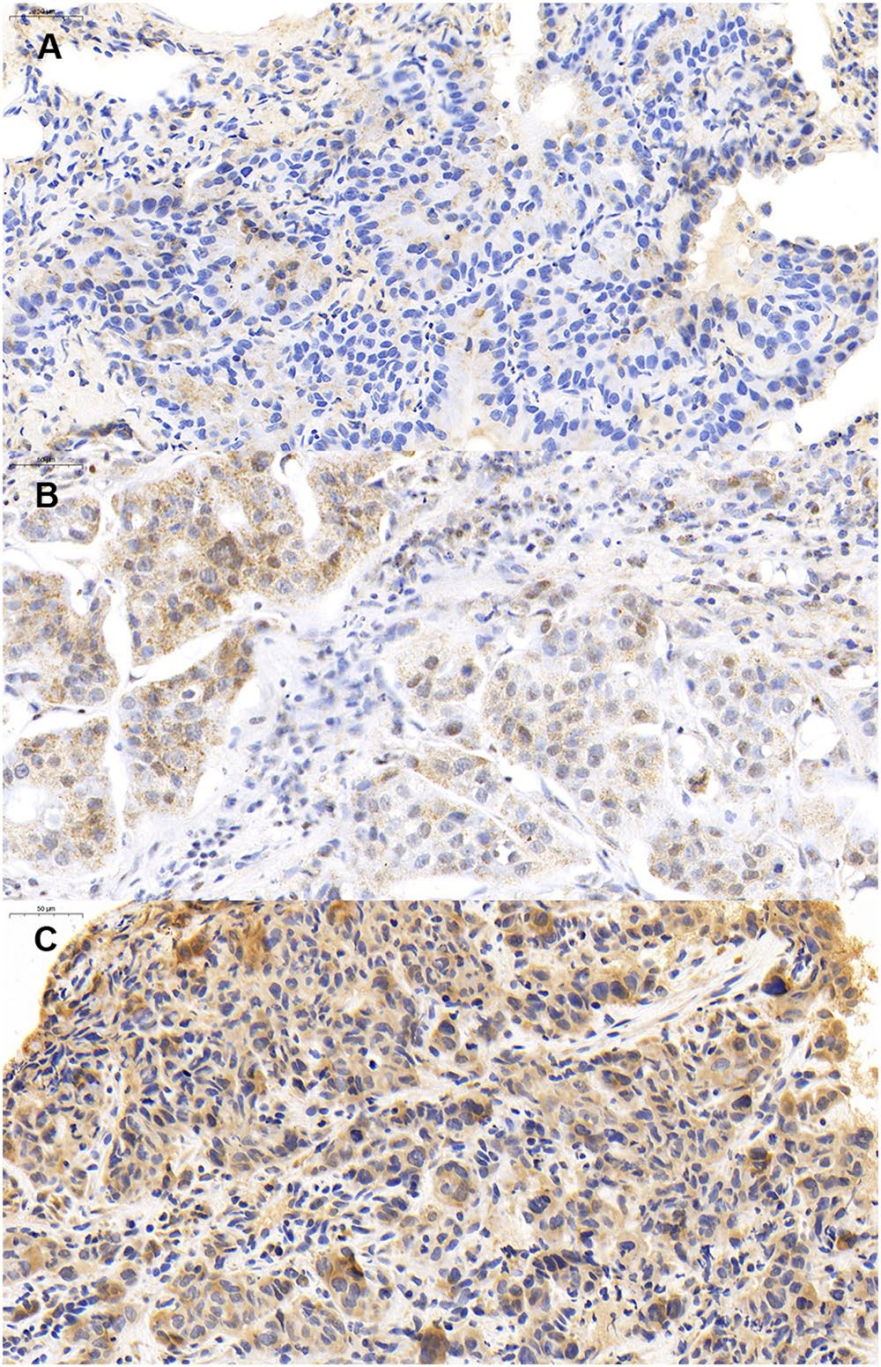
Representative immunohistochemical staining of MTAP in advanced lung adenocarcinoma. (**A**) Faint cytoplasmic IHC expression of MTAP. (**B**) Moderate cytoplasmic IHC expression of MTAP. (**C**) Strong cytoplasmic IHC expression of MTAP.

As shown in Fig. 1, MTAP was expressed to varying degrees in the cytoplasm. MTAP low expression was observed in 61.2% (101/165) of patients. MTAP expression was not correlated with gender (*p* = 0.41), age (*p* = 0.11), smoking status (*p* = 0.23), clinical T (*p* = 0.34), N (*p* = 0.70) stage, and EGFR status (*p* = 0.41) (Table 1).

### MTAP low expression was correlated with improved treatment response

Objective response rate (ORR) for the whole group is 57.6% (95/165), whereas it was 64.4% (65/101) in the MTAP-low cases and 46.9% (30/64) in the MTAP-high cases (*p* = 0.035; OR 0.49, 95% CI: 0.26–0.92). Moreover, disease control rate was higher in the MTAP-low cases (92.1% vs. 79.7%, *p* = 0.03; OR 0.57, 95% CI: 0.26–0.92). The data was detailed in Table 2.

**Table 2:** Clinical response of first-line therapy for 165 patients and associations with MTAP expression.

	MTAP-high (n = 64, %)	MTAP-low (n = 101, %)
DCR	51 (79.7)	93 (92.1)
PR	30 (46.9)	65 (64.4)
SD	21 (32.8)	28 (27.7)
PD	13 (20.3)	8 (7.9)

In addition, as shown in Table 1, a total of 79 patients experienced tumor recurrence, while 55 (54.5%) patients were in the MTAP-low expression group, in contrast to 24 (37.5%) in the MTAP-high expression group (*p* = 0.04).

### MTAP low expression predicts lower survival rate

**Figure 2:**
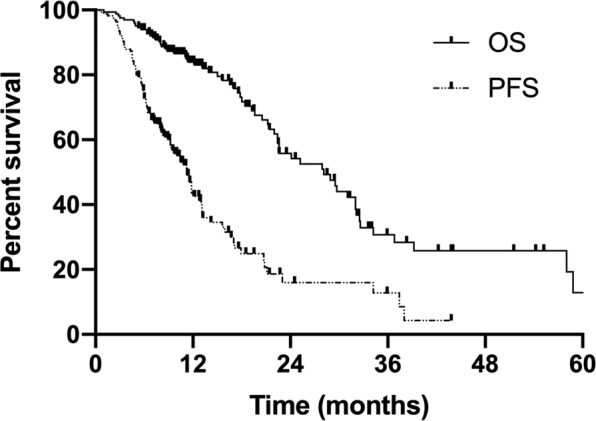
Survival curves of progression-free survival and overall survival in the whole patients with metastatic lung adenocarcinoma. The median PFS and OS were 11.3 months (range, 1.0–43.8 months) and 28.1 months (range, 1.0–70.9 months).

**Figure 3:**
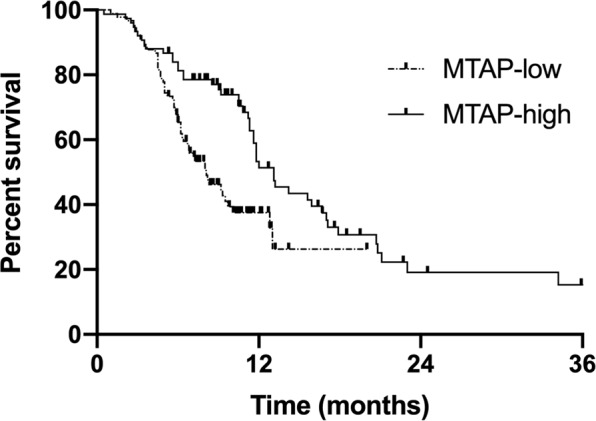
Progression-free survival curve of patients stratified by MTAP expression. The median PFS in the MTAP-low group was 8.1 months, compared to 13.1 months in the MTAP-high group (*p* = 0.002).

**Figure 4:**
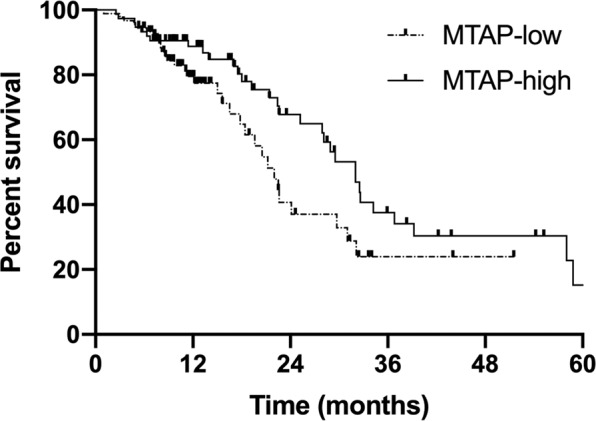
Overall survival curve of patients stratified by MTAP expression. The median OS was 22 months in the MTAP-low group, whereas it was 32 months in the MTAP-high group (*p* = 0.044). The 1- and 2-year survival were higher in the MTAP-high group (88.8% vs. 79.5%; 67.8% vs. 40.7%, respectively).

The median follow-up time was 32.6 months. To the end of the follow-up date, 41.2% (68/165) of patients were still alive. For the whole group, the median progression-free survival (PFS) and survival time (MST) were 11.3 months (range, 1.0–43.8 months) and 28.1 months (range, 1.0–70.9 months) (Fig. 2). The median PFS and MST were 8.1 months and 22 months in the MTAP-low group, in contrast to 13.1 months (*p* = 0.002) and 32 months (*p* = 0.044) in the MTAP-high group, respectively (Figs. 3 and 4). The 1- and 2-year survival rates in the MTAP-low group were 78.6% and 61.9%, respectively, which were worse versus 88.8% and 67.8% in the MTAP-high group, respectively.

In univariate analysis as shown in Table 3, gender, smoking, T/N stage, brain metastasis (BM), TRT, and MTAP expression were associated with survival. Multivariate analysis demonstrated BM (HR 1.55, 95% CI 1.02–3.41, *p* = 0.046), TRT (HR 0.52, 95% CI 0.16–0.97, *p* = 0.026) and MTAP-low expression (HR 1.36, 95% CI 1.01–4.48, *p* = 0.038) each independently predicted unfavorable overall survival (OS).

**Table 3:** Univariate and Multivariable analyses of overall survival.

Variables	Univariate analysis		Multivariate analysis	
	HR (95% CI)	*p*	HR (95% CI)	*p*
Age (≥60 vs. <60)	1.68 (1.03–4.74)	0.12		
Gender				
Male vs. Female	2.72 (1.63–8.21)	0.01	4.79 (0.81–5.23)	0.09
Smoking^a^	2.13 (1.22–5.64)	0.07	1.56 (0.83–4.52)	0.28
T stage (T1-2 vs. 3-4)	0.45 (0.19–0.68)	0.04	0.36 (0.19–4.67)	0.31
N stage (N0-1 vs. 2-3)	0.33 (0.11–0.56)	0.034	0.42 (0.28–1.53)	0.17
EGFR status				
NA	Reference			
Wild-type	0.54 (0.24–1.24)	0.15	0.48 (0.34–1.67)	0.69
Mutant	0.48 (0.20–1.16)	0.10	0.31 (0.14–2.21)	0.77
BM status				
BM vs. non-BM	1.23 (1.02–3.14)	0.028	1.55 (1.02–3.41)	0.046
First-line chemo cycles				
4 vs. 6	0.88 (0.55–1.34)	0.09	0.46 (0.13–1.62)	0.23
TRT	0.73 (0.48–0.92)	<0.01	0.52 (0.16–0.97)	0.026
MTAP expression				
Low vs. High	1.78 (1.03–3.17)	0.01	1.36 (1.01–4.48)	0.038

BM, brain metastasis; TRT, thoracic radiation. Smoking^a^: Former/current vs. never.

## Discussion

In the present study, MTAP expression was performed in tumor sections biopsied from metastatic lung adenocarcinoma patients initially treated with pemetrexed-platinum chemotherapy plus bevacizumab to explore the correlation of its expression and clinical outcomes. The results showed low MTAP was expressed in 61.2% of patients. MTAP expression was not associated with gender, age, smoking status, and T/N stage, but it was correlated with tumor recurrence. Notably, MTAP low expression was significantly associated with better ORR (64.4%, *p* = 0.035) and disease control rate (92.1%, *p* = 0.03). However, the PFS and OS were worse in MTAP-low expression group compared to that in MTAP high expression group significantly. Our results showed MTAP expression might predict the response and survival in the setting, but there are still factors left to be explored. Such factors may be explained by the mechanism of MTAP pathways and other potential correlated pathways. In addition, the complexity of subsequent clinical treatment strategies may contribute to the appearance of the contrast results.

MTAP plays a vital role in the methionine salvage pathway, which converts MTA to adenine and methionine, then the two metabolites were recycled to synthesize MTA. MTAP was encoded by the MTAP gene, which is localized in the chromosome 9p21 region, where its proximity tumor suppressor gene CDKN2A is also located. As a result, the MTAP gene is frequently co-deleted in human tumors^13^. In cancer cells lacking MTAP, purine synthase mainly depends on the *de novo* pathway due to the absence of adenine, whereas in normal cells MTA could convert to the adenine metabolite AMP (involved in *de novo* purine biosynthesis) in the presence of MTAP via the salvage pathway.

Based on the metabolic difference, blockade of *de novo* purine synthesis in MTAP-deficient tumors, in theory, might be a potent strategy to inhibit tumors without affecting normal cells. Several studies demonstrated MTAP-deficient tumor cells are more sensitive than MTAP-positive cells to purine synthesis inhibitors^7,14,15^. In breast cancer cell lines, the cytotoxic activity of 5-fluorouracil and methotrexate were increased after MTAP knockdown^15^. MTAP was often used to predict outcomes in clinical practice. In a retrospective study, 99 non-small cell lung cancer patients treated with surgery were collected to explore the correlation of MTAP and survival^16^. The result showed the patients with an MTAP-low expression had poor overall survival (*p* = 0.01) and disease-free survival (*p* = 0.002) compared to that with a high MTAP expression. To further explore the possible reasons for the opposite results, this retrospective study was conducted but conferred the conclusion that patients with low MTAP expression had worse survival. The MST in the MTAP-low group was 22 months, in contrast to 32 months in the MTAP-high group (*p* = 0.044).

The conflicting result, that is a good response but with poor survival, suggested that there may be other pathways affecting the sensitivity of MTAP-deficient tumor cells to pemetrexed. The previous study indicated that the activity of pemetrexed did not only depend on MTAP expression^17^. MT-DADMe-Immucillin A (ImmA), a highly potent transition state inhibitor of MTAP, was used to address the effect of MTAP on pemetrexed activity in two mesothelioma cell lines, NCI-H28 MTAP (+) and NCI-H2052 MTAP (−). In the presence of thymidine, the pemetrexed IC_50_ decreased significantly in H28 MTAP+ cells, which were exposed to ImmA (ImmA was demonstrated no effect on cell growth alone); conversely, transfection of MTAP into H2052 increased pemetrexed IC_50_ by nearly 3-fold. Moreover, the study proved that thymidylate synthase (TS) was the primary target for pemetrexed. Another study also revealed that the sensitivity of pemetrexed depends on the level of TS^18^. In A549 lung cancer cell line with downregulated TS level induced by docetaxel-resistance, pemetrexed sensitivity was increased compared to those parental cells, whereas it was decreased when exogenous TS was overexpressed in the docetaxel-resistant A549 cell line. Furthermore, MTAP reintroduced into MTAP-deleted HT1080 fibrosarcoma cells resulted in a variety of phenotypes and gene expression, which was involved in Wnt or other signaling pathways^19^. However, the treatment of MTAP-expressing cells with ImmA did not result in an MTAP-phenotype. All of the aforementioned studies suggested that unknown signaling pathways existed and might be changed dynamically during the treatment of pemetrexed maintenance that caused the change of sensitivity of pemetrexed to MTAP-deficient tumors.

Change of genes may contribute to the complexity of the MTAP associated treatment. CDKN2A is frequently co-deleted with MTAP, while the status of MTAP IHC expression reflected the status of CDKN2A in malignant pleural mesothelioma diagnosis^20,21^. p16^INK4A^ and p14^ARF^ are two well-studied proteins encoded by the CDKN2A gene p16^INK4A^ binds to two cell cycle-involved proteins CDK4 and mCDK6, whereas p14^ARF^ protects the tumor suppressor protein p53. Hence, the effect of concordant loss of MTAP and CDKN2A may interact with each other. Further, disordered methionine metabolism induced by MTAP/CDKN2A deletion led to dependence on protein arginine methyltransferases-5 (PRMT5), which was correlated with p53 suppressor function and involved in growth control and development^22,23^. Due to MTAP deficiency, MTA cannot be further metabolized, which makes it accumulation in the cytoplasm. A recent study indicated that the endogenous inhibitor of PRMT5 increased due to MTA accumulation and synergized the antitumor activity of the novel type I PMRT inhibitor^24^. Given the complexity of molecular signaling pathways, MTAP status alone is insufficient to distinguish the efficacy of antitumor therapeutics.

Furthermore, MTAP might play as a tumor suppressor gene in tumors, which may explain to a certain extent why the response is good, but survival is poor in MTAP-deficient tumors. The knockdown of MTAP expression promoted the invasion and migration of esophageal squamous cell carcinoma (ESCC) cells, but it was opposite while MTAP was overexpressed^25^. Further analysis indicated that MTAP deletion could promote the epithelial-to-mesenchymal transition of ESCC cells via the GSK3β/Slug/E-cadherin axis. For pediatric gliomas, MTAP were more frequent in grade IV gliomas (62.5%, *p* = 0.0087) but rare in grade I gliomas (12.2%, *p* = 0.0178); besides, MTAP gene deletion was correlated with a poor survival (*p* = 0.01) and a shorter PFS (*p* = 0.016)^26^. The results from the Ewing sarcoma family of tumors also revealed loss of MTAP expression was a negative prognostic marker^27^. The present study was consistent with previous studies, that the shorter survival (*p* = 0.044) and PFS (*p* = 0.002) were in patients with MTAP low expression, even though they were more sensitive to pemetrexed than patients with MTAP high expression.

As a retrospective study with limited sample sizes, our study did have some limitations. First, no qPCR or RNA-seq was performed to determine MTAP gene expression. Because of the insufficient tumor tissues obtained by biopsy, poor quality control of RNA extraction from formalin-fixed, paraffin-embedded samples, and RNA degradation, qPCR and RNA-seq failed to be used in the current study. Second, no detection of the status of MTAP adjacent genes was performed to explore the correlation with MTAP deficiency and further investigate the potential role of those changes. Third, not all patients were given EGFR-mutation detection. However, the patients with or without EGFR mutation between the MTAP-low or MTAP-high expression groups were well balanced. Due to gene mutations not performed after the disease progression, and some potential molecular markers not tested, all of them make it more unclear why better response for MTAP deficiency failed to transfer into better outcomes. Furthermore, the MTAP expression was not detected in paracancerous tissues and normal lung tissues, which made it impossible to compare the expression of MTAP with that in tumor tissues. Now, we are screening some lung adenocarcinoma cell lines harboring gene mutations, such as EGFR mutations, ALK rearrangement, KRAS mutations, and BRAF mutation, and other cell lines without gene mutations to detect MTAP gene and protein expression. Drug sensitivity was tested on several chemo-drugs including pemetrexed and immune checkpoint inhibitors with or without radiation therapy to explore the role of MTAP deficiency further. The preliminary results are being organized and will be reported soon. We hope to understand the potent mechanism that MTAP low expression results in worse survival in the presence of high sensitivity on drugs.

## Patients and Methods

### Patients

Between 2013 and 2018, the records of patients diagnosed with advanced lung adenocarcinoma by histopathology and imaging studies in our cancer center were retrospectively reviewed. All patients were staged according to the criteria of AJCC 7^th^. Before the initial treatment delivered, whole-body systemic evaluation was performed, including history and physical examination, blood profile, cervical ultrasound or computed tomography (CT), chest and abdomen enhanced contrast CT, brain enhanced CT or magical resonance imaging (MRI); positron emission tomography (PET)-CT was not routinely performed in our cancer center and only given in partial patients. Patients with severe comorbidities that negatively impact on antitumor treatment or with other cancer history were excluded. In addition, patients without complete medical records were also excluded.

This study was approved by the Institutional Review Board of Shandong Cancer Hospital. All procedures performed in studies involving human participants were in accordance with the 1964 Helsinki declaration and its later amendments or comparable ethical standard. All patients and/or legal guardians signed informed consent forms before inclusion in the present study and for future study.

### Treatment strategies

All patients were treated with pemetrexed (500 mg/m^2^, day 1) plus cisplatin (75 mg/m^2^, given in 3 times with 25 mg/m^2^ per time, day 1, 2, and 3) or carboplatin (AUC = 5) per 3 weeks up to 6 cycles. Folic acid, vitamin B12, and dexamethasone were given as clinical routine. Bevacizumab was delivered at day 0 with a dosage of 7.5 mg/kg and was also given every 3 weeks up to 4–6 cycles parallelly with chemotherapy. Treatment response was evaluated according to the Response Evaluation Criteria in Solid Tumors version 1.1. For patients with stable disease (SD), partial response (PR) and complete response (CR), pemetrexed (500 mg/m^2^, day 1) ± bevacizumab (7.5 mg/kg, day 0) maintenance were recommended to give monthly. In addition, for patients with the non-progressive response after front-line therapy, thoracic radiation (TRT) was recommended with a dosage from 50 Gy–60 Gy in 25–30 fractions.

### Immunohistochemistry

As the previous study reported immunohistochemistry (IHC) is a reliable method to detect MTAP expression in lung cancer^28^, IHC was therefore performed to investigate MTAP expression in the present study. Tumor tissues obtained by biopsy were retrieved for the determination of MTAP expression. Formalin-fixed, paraffin-embedded samples of 4uM thickness were air-dried overnight at room temperature. Then sections were deparaffinized in xylene and rehydrated in graded alcohol. Antigen retrieval was performed for 60 min with pH 8.0 TRIS-EDTA buffer. The primary polyclonal rabbit anti-human MTAP antibody (1:500; Thermo Fisher Scientific, US) and secondary antibody were applied to stain the slides. The slides were subsequently incubated in diaminobenzidine for color detection. Finally, the sections were counterstained with hematoxylin, dehydrated and sealed with a coverslip.

IHC staining assessment of all samples was performed independently by two pathologists who were blind to patient clinical information. Only cytoplasmic MTAP expression of tumor cells was evaluated on immunoreactivity intensity and percentage. The staining intensity was scored as 0 (no staining), 1 + (weak), 2 + (moderate), and 3 + (strong). The percentages of positive staining tumor cells in 3 randomized fields was then calculated and scored as 0 (0–5%), 1 (6–25%), 2 (26–50%), 3 (51–75%) and 4 (>75%). The final immunohistochemical score was the product of the score of the proportion of positive staining tumor cells and the staining intensity score, which defined as faint staining (0–4), moderate staining (5–8) and strong staining (9–12). MTAP low expression was defined as the final score 0–4.

### Statistical analysis

SPSS 23.0 was used to perform statistical analysis (SPSS, Armonk, NY: IBM Corp). Chi-square test or Fisher’s exact test was used to evaluate the association between clinical features and MTAP expression. PFS and OS were measured from the date of initial treatment to the event occurrence or the last known date of follow-up. Kaplan-Meier method was performed to calculate PFS and OS, and the log-rank test was used to evaluate the difference in survival curves between different groups. Cox proportional hazards regression model was used to calculate hazards ratios (HR) with 95% CI for survival. All statistical tests were two-sided, and *p* < 0.05 was considered statistically significant for all analyses.
